# The role of benzydamine in prevention and treatment of chemoradiotherapy-induced mucositis

**DOI:** 10.1007/s00520-021-06048-5

**Published:** 2021-03-01

**Authors:** Ourania Nicolatou-Galitis, Paolo Bossi, Ester Orlandi, René-Jean Bensadoun

**Affiliations:** 1 Dental School, National and Kapodistrian University of Athens, Athens, Greece; 2 Department of Medical and Surgical Specialties, Radiological Sciences and Public Health-Medical Oncology, University of Brescia, ASST-Spedali Civili, Brescia, Italy; 3 National Center of Oncological Hadrontherapy (CNAO), Pavia, Italy; 4 Centre de Haute Energie, Nice, France

**Keywords:** Mucositis, Radiation therapy, Chemotherapy, Oral mucositis, Benzydamine, Anti-inflammatory agents

## Abstract

**Purpose:**

To discuss *the role of benzydamine* in the prevention and treatment of radiation-induced oral mucositis (OM) in head and neck (H&N) cancer patients. This document represents an *expert opinion paper* on indications and key-role aspects in *OM pathogenesis*, *prevention and treatment*.

**Oral mucositis:**

OM represents a common side effect of chemotherapy (CHT) and radiotherapy (RT). It consists in a painful erythema involving the oral cavity mucosa, which may progress to ulceration. Five biologically dynamic phases are considered crucial in mucositis: “initiation, signalling, amplification, ulceration and healing”. Oral environment and microbiota are fundamental in mucositis development being involved in susceptibility to infections and in ulceration consequences. Different agents against mucositis have been studied and the use of benzydamine is strongly supported in literature. The Multinational Association of Supportive Care in Cancer and International Society for Oral Oncology (MASCC/ISOO) guidelines recommend its use for the prevention of OM in H&N patients undergoing RT and RT/CHT.

**Benzydamine:**

Benzydamine is a local anti-inflammatory drug with analgesic properties. It can decrease TNF-α, IL-1β and prostaglandin synthesis, also inhibiting leukocyte-endothelial interactions, neutrophil degranulation, vasodilation and vascular permeability. Literature agrees on the beneficial effects of benzydamine in preventing and reducing oral mucositis severity in H&N cancer patients undergoing RT/CHT.

**Conclusions:**

Mucositis represents a major concern in H&N cancer patients and a clinical and economical issue. A multimodal and multidisciplinary approach is needed for its management. International guidelines recommend benzydamine for OM prevention and treatment in H&N cancer patients, but further “real world” trials should be designed.

## Introduction/Background

Mucositis represents a common side effect of antineoplastic treatments like chemotherapy (CHT), radiotherapy (RT) and hematopoietic stem cell transplantation (HSCT). Preventing and minimizing this treatment-related adverse event may lead to achieve better treatment intensity not compromising patient’s quality of life.

**Fig. 1:**
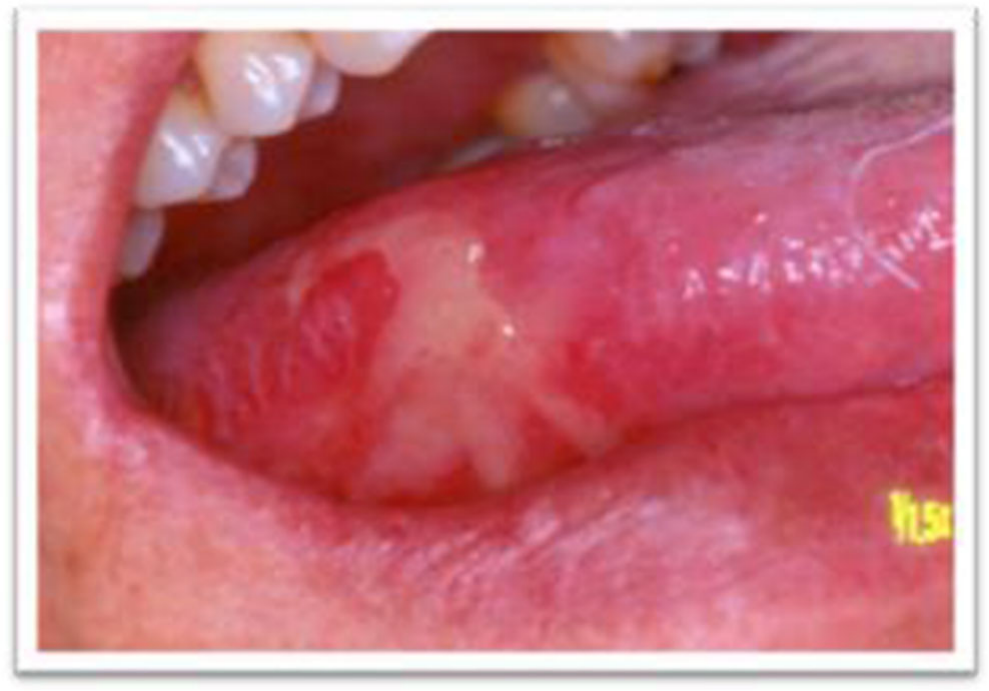
Oral radiation-induced mucositis, lateral tongue. Ulcers on non-keratinized mucosa (*Courtesy of Nicolatou-Galitis*)

Oral, pharyngeal and gastro-intestinal (GI) mucositis is mainly characterized by an inflammation of the mucosa caused by cancer treatments that lead to an important damage causing pain, difficulty in eating and swallowing and diarrhoea. All GI tracts can be affected by mucositis. Oral mucositis is reported to occur in about 40% of patients receiving CHT and in 80% of patients undergoing head and neck (H&N) cancer RT with or without CHT [1]. Oral mucositis (OM) involves the oral cavity and typically presents as a painful erythema, which progresses to mucosal ulceration impairing nutritional intake and patients’ quality of life [2]. OM can be the most debilitating side effect in patients with head and neck cancer who receive radiotherapy or chemoradiotherapy (Fig. 1). Severe OM may develop in 35 to over 60% of head and neck cancer patients and may not be relieved by opioid analgesia. Radiation-induced oral mucositis has also a significant economic impact due to costs associated with pain management, liquid diet supplements, gastrostomy tube placement or total parenteral nutrition, management of secondary infections and hospitalizations. OM has an interplay with oral infections. Candidiasis and herpes simplex virus-1 infections have been reported to develop and be superimposed on radiation mucositis [3–5]. Oral candidiasis develops with an incidence of 27 to 52.5%, and herpes simplex virus infection may develop in more than 20% of patients during radiotherapy. A proper differentiation of oral infection from mucositis is necessary for a successful diagnosis and management of OM (Table 1).

**Table 1:** Clinical criteria for easy differential diagnosis in mucositis

Oral mucositis	Oral pseudomembranous candidiasis	Herpes simplex infection
Ulcers on the non-keratinized mucosa (lateral and ventral tongue, buccal and labial mucosa, soft palate)	Any area of oral mucosa can be involved	Ulcers on the keratinized mucosa (masticatory mucosa: fixed gingival, hard palate, dorsum of tongue)
Patient will not let us “remove pseudomembranes” They are painful	We can remove the pseudomembranes easily They are not painful	Patient will not let us “remove pseudomembranes” They are painful

OM severity is directly linked to the total dose and fractionation of the radiation treatment and, especially, to its association with CHT [6]. Moreover, patients experiencing RT-CHT-related mucositis might need treatment unplanned breaks or total planned dose reduction thus risking a negative impact both on treatment outcome and on survival rates [2].

In the recent years, several predictive multivariable models including inpatient-, tumour- and treatment-related factors have been developed in order to implement clinical support to the patients at higher probability of developing severe OM [7, 8].

Even if a large series of different agents against mucositis have been studied, preventive and therapeutic options for OM having a high level of evidence are quite limited [9]. The lack of well-conducted clinical trials, limited resources given to research about mucositis and the misleading concept that investments in oncological drugs are more important than in supportive care may explain this discrepancy.

Basic oral care is recognized as being critical in OM prevention, and bland rinses increasing oral clearance are considered helpful for maintaining oral hygiene and improving patient comfort [10]. The use of other agents with anti-inflammatory properties has no huge literature in its support, except from benzydamine, which role is underpinned in scientific literature.

## Paper objectives

The *objective of this publication* is to discuss *the role of benzydamine* in the prevention and treatment of radiation induced OM in H&N cancer patients in order to further detail the features of this drug in a more complete and extensively multidisciplinary context.

The *present document represents* the expert opinion paper on the evidence that endorse both management indications and key-role *aspects in mucositis pathogenesis*, *prevention and treatment*.

## Mucositis

### Mucositis pathophysiology

Historically, mucositis has been identified as the simple result of RT/CHT-induced apoptosis in rapidly dividing basal epithelial stem cells.

Thanks to the efforts made in this field of research, we know, now, that it does not represent only the direct result of clonogenic cell death but it embodies the outcome of a sequence of complex biological events, interacting with the underlying treatment-related mucosal injury [11, 12].

These findings have, undoubtedly, provided changes in prevention and treatment approaches to OM by creating the opportunity to act on different pathways.

Five phases have been identified as crucial for the pathophysiological progression of mucositis, and the whole process is considered biologically dynamic [13].

The “initiation phase” is basically characterized by an RT-CHT-induced DNA damage that, after causing a double-strand break, results in the basal epithelial cells clonogenic death. This process leads to the production of reactive oxygen species (ROS). In an early response phase, a wide number of transcription factors are activated thanks to the initiation of transduction pathways triggered both by DNA injury and by lipid peroxidation [14]. This is the moment in which the “signalling and amplification phases” start. The most investigated signal pathway is the NF-kB because it easily represents the pathobiology of OM. NF-κB is activated to upregulate a series of genes in the endothelium, fibroblasts, macrophages and epithelium, leading the production of messaging and effector proteins, such as pro-inflammatory cytokines, stress responders and cell adhesion molecules [11, 12]. Pro-inflammatory cytokines regulated by NF-κB, such as IL-6, IL-1β and TNF-α, account for an important mechanistic component in the pathogenesis of mucositis. The protein levels of these cytokines in both tissue and peripheral blood are positively associated with the severity of mucosal toxicity [15]. Cytotoxic therapies are able to work even on the connective tissues by inducing fibrinolysis which, through the stimulation of macrophages, leads to the production of metalloproteinases [12].

All these molecules may positively or negatively feedback on different pathways thus guiding local tissue response. These complex mechanisms occur simultaneously and through a series of heterogeneous networks [10]. For example, TNF-α usually amplifies NF-kB response and activates mitogen-activated protein kinase (MAPK) signalling.

Once the inflammatory cascade is activated, its progression leads to the “ulceration phase” which is considered the major mucositis event as it causes pain, loss of appetite and, sometimes, patient hospitalization. It is the consequence of direct and indirect injury causing damages and apoptotic changes both in the epithelial cells and in the connective tissues. Ulcers can be easily colonized by oral bacteria, which extend and worsen the entire mucositis process.

An inflamed submucosa, rich in macrophages, receives cell wall products as lipopolysaccharides (LPS) and cell wall antigens that further stimulate the production of pro-inflammatory cytokines thus augmenting mucositis symptoms. When treating neutropenic patients, it has to be considered that in case of bacteria invading submucosal vessels, bacteremia or sepsis can be observed [12].

Fortunately, most of the patients suffering from OM recover spontaneously within approximately 2–4 weeks after the last dose of stomatotoxic chemotherapy or radiation therapy. The “healing phase” comprises all the active biological mechanisms that lead the process towards the resolution of ulcers. The process starts in the submucosa extracellular matrix (ECM) where signalling triggers the migration and differentiation of the epithelium that heals the ulcer. Furthermore, ECM impacts on healing by activating intrinsic tyrosine kinase; it modulates receptors and the expression, organization and activation of intracellular proteins [12].

From what is being explained above, it appears that there is a lag between molecular/cellular damage and clinical manifestations. In patients undergoing fractionated radiation treatments, daily dose increments trigger mucositis development. For patients treated with chemotherapy, mucositis is, instead, an acute event, with symptoms occurring generally 3–5 days after drug administration. Ulcers usually appear in 7–9 days, and often heal in 2 weeks [12].

### Factors influencing mucositis development

Oral environment and microbiota are considered of crucial importance in the development of mucositis.

The oral cavity is one of the most complex environments in the human body. Saliva, constantly produced by salivary glands, contains bacteria, fungi and viruses, and moistens every part of the oral mucosa. A dysbiotic milieu may alter the metabolism of the host and determine increased inflammation [16].

Studies in literature based on animal models demonstrated that a peak in bacterial loads coincided with a peak in mucositis severity [12]. Moreover, other studies showed that higher concentrations in Gram-negative bacteria were observed during ulceration phases, and spontaneous ulcer resolution happened after a reestablishment of normal bacterial proportions [17].

Recently, a different way of looking at microbiota is emerging, showing that there is a complex interaction between the host and diverse bacterial species.

In this reality, treatment-related dynamic changes are important both in oral and intestinal mucositis [18].

Studies conducted in the last years focused on different topics: oral flora changes in chemotherapy regimens [19] and its relationship with oral mucositis; microbiota impacts on healing process [20]; and functional changes in bacteria when exposed to radiation [21] and their impact on mucositis. In this field, rapid advances have also been achieved in the area of intestinal mucositis [22]. A handful of papers have been published also for oral and oropharyngeal mucositis [23, 24]. The dynamic variation in oral microbiota during RT and its association with oropharyngeal mucositis progression/aggravation were prospectively evaluated in a cohort of nasopharyngeal cancer patients. Several bacteria, including *Prevotella*, *Fusobacterium*, *Treponema* and *Porphyromonas*, not only showed obvious dynamic synchronous shifts in their abundances throughout RT, but their peaks often coincided with the onset of severe mucositis [25]. Similar results were reported by Zhu et al. [26]. Two studies only investigated on oral microbiota treatment-related changes and identified species potentially involved in mucositis pathogenesis [27, 28].

Thanks to the evidence showing overlapping elements between animal models and humans in the field of microbiota, different studies have been performed suggesting a general decrease in microbial diversity after the completion of antineoplastic treatments [29–31], and an increased in relative concentration of Proteobacteria and anaerobes (*E. coli* and *Salmonella* spp.) [30–33].

In general, there is a sort of global agreement on the fact that oral microbiota plays an important role in this setting of patients, being involved both in the susceptibility to infections and in the consequences of the ulceration phase. Anyway, it seems importantly affected and altered by cancer treatments but understanding on the mechanism of this process still remains poor [27].

In this area, further studies are needed to clarify the complex relationship between oral microflora and mucositis in order to consider microbiota’s role in all the phases of mucositis pathophysiology, rather than viewing it as a passive contributor to the ulcerative phase.

## Multifactorial approach and international guidelines

As soon as inflammation has been widely demonstrated to play a key role in the pathogenesis of mucositis by initiating a broad of cascades and pathways that leads to ulceration, studies have concentrated their efforts in finding the best way to prevent and treat this common RT and CHT side effect. Through the years, lots of strategies have been proposed to control both the initiation and progression of mucositis. Anti-inflammatory agents, analgesics, mouthwashes, oral care protocols and other non-pharmacological remedies and devices (photobiomodulation for example) are reported in literature as being the historical milestones when approaching patients suffering from mucositis.

In everyday clinical practice, all these agents and strategies are usually combined in a markedly different way.

The Multinational Association of Supportive Care in Cancer and International Society for Oral Oncology (MASCC/ISOO) guidelines provide clinicians with a set of interventions for mucositis based on strong evidence. They are continuously updated through a systematic approach to weigh the evidence and analyze the clinical applicability of different strategies [9, 34–36].

Mucositis management is a complex objective because of the presence of many confounding factors; thus, a multimodal combined methodology should be considered and strongly preferred. Agents, both local and systemic, able to address the multifactorial aspects and multi-layered basis of mucositis are robustly recommended. Moreover, both initiation and progression of mucositis are deeply influenced by the patients’ different baseline characteristics like microbioma, oral hygiene and saliva environment. The optimal treatment should, thus, be designed both on patients’ characteristics and on the idea that mucositis needs, definitely, an extensively multimodal strategy based on the contribution of a multidisciplinary team supporting patients in every phase of their treatment. This team should comprise other professional stakeholders like dentists, nutritionists, pain therapists and nurses in order to tailor treatment on patients thus considering the patient as a whole.

The MASCC/ISOO newly updated guidelines provide a set of recommendations and suggestions for the prevention and treatment of mucositis. The panel suggests the use of a multi-agent combination in the basic oral care (BOC) section for patients undergoing RT, CHT and HSCT and recommends the use of benzydamine, with a level of evidence I, for the prevention of OM in patients receiving a moderate RT dose (<50 Gy) for H&N cancer. The drug is even suggested for OM prevention in patients undergoing combined RT/CHT. Photobiomodulation (PBM) is a fundamental element in the multifactorial approach of OM. It is based on the use of an intraoral (or trans-cutaneous) low-level laser therapy device, in red or infra-red wavelength, with a specific dose and rigorous physics parameters. The panel recommends its use for mucositis prevention both in adult patients undergoing HSCT conditioned with high dose CHT and in patients with H&N cancer receiving RT (given alone or in combination with CHT). Cryotherapy is recommended mainly in HSCT when the conditioning includes high doses of melphalan and in patients undergoing systemic treatments including 5-FU boluses [9].

## Benzydamine

Benzydamine is a local anti-inflammatory drug with analgesic and anaesthetic properties. It recalls NSAIDS’ activity; however, benzydamine works on local inflammation factors not influencing systemic physiological mechanisms. It has been importantly investigated because of its capacity both to interact with different inflammation pathways and to work on inflammation and pain [37–43].

Recently recommended in the new MASCC/ISOO guidelines [9], it represents, nowadays, one of the most important agents in RT-related mucositis prevention. Benzydamine (N,N-dimethyl-3-[(1-benzyl-1H-indazol-3-yl)ossi]-1-propanamine) is a non-steroidal anti-inflammatory drug extensively recognized, both in clinical practice and in international guidelines, as a landmark in the topical treatment of mucositis associated with RT. It has been shown that benzydamine plays a suppressive role in the production of proinflammatory cytokines, since it inhibits TNF-α production. This fundamental topical activity turned on the lights on this drug giving birth to different clinical studies that investigated its role in mucositis prevention and treatment.

Benzydamine mechanisms of action reside in its anti-inflammatory, anaesthetic and analgesic nature. It has been demonstrated that it may act both by decreasing TNF-α, IL-1β and prostaglandin synthesis and as an antioxidant (ROS scavenger). It is able to inhibit leukocyte-endothelial interactions thus reducing neutrophil degranulation capacity and it works on reducing histamine-induced vasodilation and vascular permeability.

A study by Sironi demonstrated that benzydamine is able to inhibit inflammatory cytokines production in human and murine mononuclear phagocytes (PBMC) exposed to different triggers in vitro. This finding was observed mainly on TNF-α and in IL-1β, which were basically less affected than the former in human models. While both TNF-α and IL-1β declined, IL-6 and IL-8 levels resulted unaltered. Moreover, in an in vivo setting, benzydamine was able to inhibit the glycolipid-lipopolysaccharide (LPS) complex lethality in mice and to reduce blood levels of TNF-α and IL-1β [38].

Another study by Sironi extended and confirmed the results about the inhibition of proinflammatory cytokines produced by benzydamine. It was observed that in PBMC taken from a normal donor and exposed to LPS, benzydamine caused a dose-dependent inhibition of TNF-α production while it did not affect the anti-inflammatory cytokines IL-10 and IL-1ra levels. These results confirmed that the anti-inflammatory activity of benzydamine is carried out by the selective inhibition of pro- versus anti-inflammatory cytokines [39].

Moreover, two systematic reviews published in 2013 [44] and in 2019 [45] and five research articles [37, 40–43], performed between 2001 and 2017, on the use of benzydamine to prevent or manage oral mucositis in H&N cancer patients receiving radiotherapy, are available (Table 2).

**Table 2:** Clinical trial and reviews characteristic and results

Authors/year/number of patients	Type of research	Type of cancer	Mucosal areas assessed/particular exclusions	RT dose, Gy	KPS/ECOG	Benzydamine administered	Effect of Benzydamine
Epstein et al./2001/patients: 160	Multicenter/randomized/double-blind/Placebo-controlled	Head-neck carcinoma/ oral, lip, pharynx: 78% larynx, saliv. glands, others: 22%	All, including hard palate, gingivae, dorsum of tongue. *Candidiasis was diagnosed*	50 Gy	>80%	Prior to start RT to 2 weeks after completion	Significant reduction of oral mucositis and oral analgesics
Cheng et al./2006/ patients: 14	Prospective/Randomized/Double-blind against Chlorhexidine	Nasopharyngeal/non-nasopharyngeal, including saliv. glands, etc.	Not reported. *Mucosal infections not assessed*	66 to 68 Gy	-	From 1st day to 2 weeks after completion	Trend of reduction of oral mucositis, pain and dysphagia
Kazemian et al./2009/patients: 81	Prospective/randomized/double-blind/placebo-controlled	Head-neck carcinoma, not otherwise specified	Hard palate, oral tongue, oropharynx, buccal mucosa, floor of mouth. *Nystatin prescribed when mucositis > grade 2*	61.69 Gy, mean	>70%	A day from start to end of RT	Significant reduction of oral mucositis
Roopashri et al./2011/patients: 100	Prospective/placebo-controlled against chlorhexidine and povidone iodine	Head-neck carcinoma, not otherwise specified	Not specifically defined/excluded ages >30y and over 79y, with bacterial/fungal infections, mucosal changes, dryness, receiving antibiotics, analgesics	66 Gy	-	After 2 weeks of start of RT at onset of mucositis	Benzydamine more efficient to delay mucositis and reduce intensity of pain
Rastogi et al./2016/patients: 120	Prospective/randomized/controlled RT or ChemoRT groups against saline	Head-neck carcinoma, not otherwise specified	Not specifically defined/oral infections not assessed	>60 Gy	>70	Not clear	Significant reduction of oral mucositis in RT
Systematic reviews on benzydamine effect on oral mucositis							
Nicolatou-Galitis et al./2013/MASCC/ISOO systematic review			Benzydamine is recommended for prevention of oral mucositis in H/N RT receiving moderate-dose radiation therapy up to 50 Gy, without concomitant Chemo				
Ariyawardana et al./2019/ MASCC/ISOO systematic review			Benzydamine mouthwash is recommended for prevention of oral mucositis in H/N RT receiving moderate-dose radiation therapy up to 50 Gy Benzydamine mouthwash is suggested for the prevention of oral mucositis in patients with H/N cancer receiving RT and CHT				

In the first systematic review published by an expert panel in 2013, the role of benzydamine was accurately reviewed both in prevention and in treatment of patients undergoing RT and CHT for H&N cancer. Six studies were reviewed, and the panel agreed in recommending the use of benzydamine mouthwash for prevention of OM in patients treated on the H&N region with moderate RT doses (up to 50 Gy) without CHT. The article reported a significant analgesic activity and symptoms amelioration observed in a double-blind and placebo-controlled study after administration of benzydamine for the treatment of OM in patients receiving RT. Although pain assessment was the primary endpoint and the study results were positive, they were judged to be inadequate for a guideline update [44].

The second review paper, published in 2019, analyzed literature for anti-inflammatory drugs used for prevention and treatment of OM in patients receiving RT, CHT, RT-CHT and HSCT. The paper reviewed different agents cited as being effective in OM and concluded that benzydamine mouthwash was the only agent with a level II evidence on its effectiveness in preventing RT-CHT-induced OM. No guideline was possible for benzydamine in settings like treatment of RT-induced OM and prevention of CHT-related OM [45].

The use of benzydamine was also investigated in five prospective and controlled clinical trials that reported significantly positive results in the use of this drug in H&N cancer patients receiving stomatotoxic treatments.

In their multicentre, randomized, double-blind, placebo-controlled clinical trial, Epstein et al. reported that benzydamine oral rinse was effective against OM in a variety of RT fractionations except for the hypofractionation (single daily dose greater than 2.2 Gy). They even stated that, up to 25 Gy, mucosal ulcers developed both in benzydamine and placebo group. Beyond this threshold, the OM distribution diverged in favour of benzydamine and a statistically significant reduction of mucositis prevalence was observed in the benzydamine group compared to placebo. Moreover, up to 50 Gy, mucosal ulceration diffusion in the benzydamine group was, in all cases, less severe than in the placebo group [37].

In a second study by Roopashri et al., in which benzydamine and other drugs were investigated, no statistically significant difference in the number of patients experiencing mucositis between the placebo group and the study groups was observed. Benzydamine oral rinse, however, reduced the intensity and duration of oral mucositis during RT. Moreover, benzydamine was considered effective not just in delaying progression of mucositis severity but also in reducing pain intensity [40].

Kazemian et al., in another double-blind placebo-controlled randomized clinical trial on the use of benzydamine in OM prevention, confirmed that benzydamine produced a statistically significant reduction in mucositis severity during RT on H&N cancer patients. An increase in the grade of mucositis both in placebo and in the study group was observed, in the first 3 weeks of treatment, reaching grade 3 at the end of RT in the placebo group, while a maximum score of grade 2 in the benzydamine group (*P* < 0.001) was observed. The authors concluded that the benefit of benzydamine on lowering the progression of mucositis to higher grades can be best observed after about 4–5 weeks from the start of RT and chemoradiation [41].

The role of benzydamine in patients undergoing RT with or without CHT was investigated also by Rastogi et al. in another clinical study. The paper showed that in patients treated with higher RT doses (>50 Gy), benzydamine was able to reduce grade 3 mucositis rates in a statistically significant manner [43].

A pilot study by Karis Kin-Fong Cheng on the use of chlorhexidine and benzydamine oral rinses in the prevention and treatment of OM, was consistent with the previously discussed works, and reported a decreasing trend in the severity of oropharyngeal mucositis in patients receiving benzydamine compared with the ones having chlorhexidine. Moreover, benzydamine delayed the onset of higher grades mucositis compared with chlorhexidine, confirming that benzydamine shows its best when used prophylactically [42].

Each of the above-mentioned studies presents some weaknesses. It has been shown previously that OM is related to the RT doses delivered during treatment, but the anatomical site involved in the RT field plays an important role in the development of OM. For example, oral mucositis is classically seen on areas of non-keratinized mucosa, with ulcers covering lateral and ventral tongue, buccal and labial mucosa and soft palate. Oral mucositis ulcerations are rarely seen on areas with keratinized mucosa, such as the hard palate and the dorsum of the tongue. Some studies try to compare mixed populations of cancer patients who may be at different risk of developing OM, depending on the irradiated mucosal areas.

Similarly, the use of benzydamine in patients undergoing concomitant chemotherapy needs to be studied more in depth. Patients should receive the same dose and schedule of concurrent systemic agent.

Moreover, in the previously cited studies, oral infections were not described or diagnosed/differentiated from oral mucositis and the concomitant use of steroids was not fully assessed.

Furthermore, timing in benzydamine administration among the cited studies varies. Benzydamine administration is initiated prior to RT or one day or even 2 weeks after RT initiation (Table 3).

**Table 3:** Summary of the characteristics of the clinical studies on the use of benzydamine: strengths and weaknesses

Clinical trials assessed (5)	Epstein (2001)	Cheng (2006)	Kazemian (2009)	Roopashri (2011)	Rastogi (2016)
Strength prospective/con-trolled studies	Yes	Yes	Yes	Yes	Yes
Weaknesses	Mucositis recorded on keratinized mucosa (minimal mucositis risk)	Oral mucosal infections with similar presentation, ulcers and pseudo-membranes not differentiated	Specific types of carcinomas, laryngeal or parotid, were not described separately	Benzydamine was used either prior or after 2 weeks of RT initiation	Real-world situation was questionable in some studies (several exclusions)
Systematic reviews (2)	Nicolatou-Galitis (2013)	Ariyawardana (2019)			
Overall Conclusion on the use of benzydamine to prevent oral mucositis		Benzydamine mouthwash is recommended for prevention of oral mucositis in head/neck cancer patients, who receive moderate-dose radiation therapy up to 50 Gy and it is suggested for the prevention of oral mucositis in patients with head/neck cancer receiving radiotherapy and chemotherapy			

Despite all the weaknesses discussed, it is important to note that all the studies agree on the positive and beneficial effects of benzydamine in preventing and reducing the severity of oral mucositis in patients affected by H&N cancer undergoing RT.

## Conclusions

Mucositis is one of the major concerns both for clinicians and for patients undergoing H&N cancer radiotherapy or chemoradiotherapy and represents a major clinical and economical issue. Over the years, multiple efforts have been made to address, prevent, delay and control the progression of OM. Different suggestions and recommendations have been published in order to better manage this common and quality of life-impairing side effect. For the next future, a multimodal and multidisciplinary comprehensive assessment might be the best choice for the prevention and management of mucositis. Multidisciplinary teams involving oncologists, and other professional stakeholders like dentists, nutritionists, pain therapists and nurses should cooperate in following patients throughout the whole course of their cancer treatment. Moreover, well-educated patients, families and caregivers will help in building patients’ compliance to treatments.

Literature findings strongly emphasize and encourage the use of benzydamine in preventing and treating OM in patients undergoing RT and CHT, but further “real world” trials should be designed in order to address all the observations on the weaknesses of the previously published studies.

## Data Availability

Yes
